# Supplementary material for: Catalytic Sabatier Process under Thermally and Magnetically Induced Heating: A Comparative Case Study for Titania-Supported Nickel Catalyst
Source: Nanomaterials (Basel). 2023 Apr 26;13(9):1474. doi: 10.3390/nano13091474 (PMC10180227; doi:10.3390/nano13091474)
Supplement: Supplementary file 1 [file nanomaterials-13-01474-s001.zip › nanomaterials-2343886-supplementary.pdf]

<u>**Electronic Supplementary Information**</u>

Prcoessing of the M-H curve

It was observed that at a low magnetic field (<1.5kOe), the hysteresis loop closes. Although, the magnetic moment does not saturate at the higher magnetic field. This behavior indicates the possible contribution from a paramagnetic component. Hence a linear extrapolation was applied to obtain the spontaneous magnetization value arising from the ferromagnetic components (figure S1).

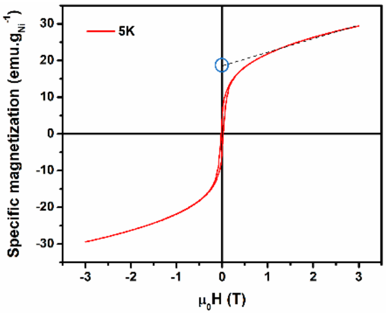

Figure S1. Determination of the spontaneous magenetization (blue circle).

Table S1. Ni nanoparticles size of the 5Ni/TiO_2 catalyst before and after catalytic test

**Samples**	**Particles size (nm) from TEM**[b]
5Ni/TiO_2 as prepared	6.7±1.7
5Ni/TiO_2 150mg	7.4±1.4
5Ni/TiO_2 300mg	7.5±1.2
5Ni/TiO_2 + H_2	7.8±1.9
5Ni/TiO_2 + SiC + H_2	7.4±1.8
5Ni/TiO_2 + SiC + H_2 stability	7.1±1.3
5Ni/TiO_2 300mg (Mag. Heating) [a]	7.3±1.4
5Ni/TiO_2 + SiC + H_2 (Mag. Heating) [a]	7.2±2
5Ni/TiO_2 + SiC + H_2 stability (Mag. heating) [a]	7.4±1.5

[a]Catalytic reaction was carried out under magentically induced heating condition.

[b]Particles size was calculated from the histogram plotted using BFTEM images of the sample.

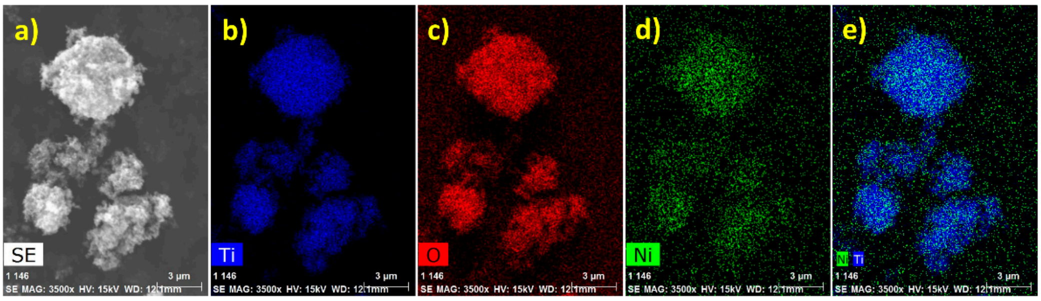

Figure S2. a) SEM-EDS image of 5Ni/TiO_2 and the EDS elemental mapping of the respective elements present in the sample, b) Ti (blue), c) O (red) d) Ni (green), and e) superposition of Ni and Ti maps.

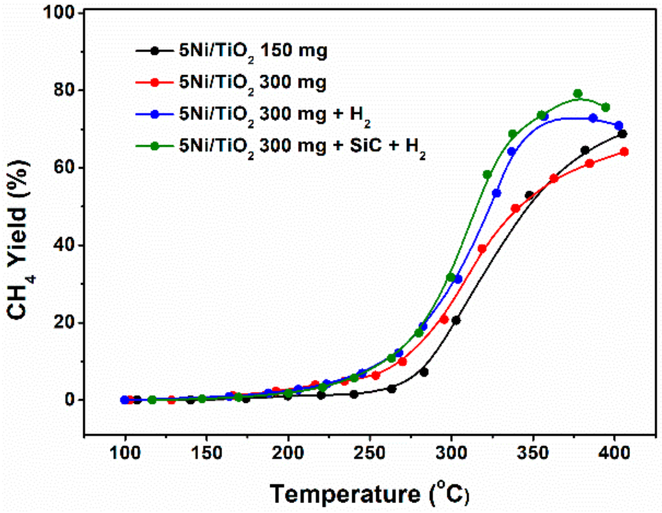

Figure S3. %CH_4 yield plotted with respect to the reaction temperature under classical heating condition.

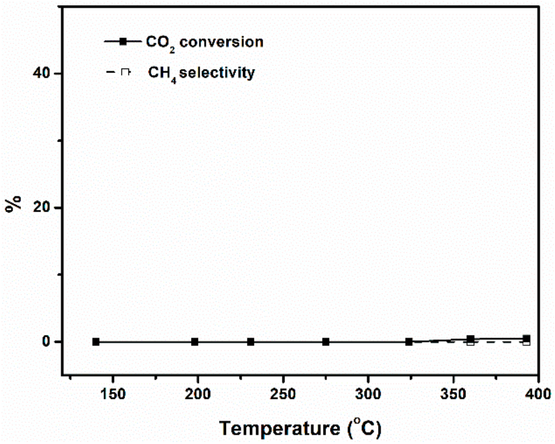

Figure S4. The $\%CO_2$ conversion and the $\%CH_4$ selectivity with respect to the temperature for pure Fe wool (300 mg). Although, pure Fe wool does not catalyse the CO_2 methanation reaction, a negligible amount of CO (<1%) was observed at around ~390˚C via reverse water gas shift reaction (RWGS). It was reported in the literature that, Fe is a potential catalyst for RWGS reaction [1,2]. In the presence of $5Ni/TiO_2$ catalyst, the Fe wool is therefore acting as a heating agent only during CO_2 methanation reaction.

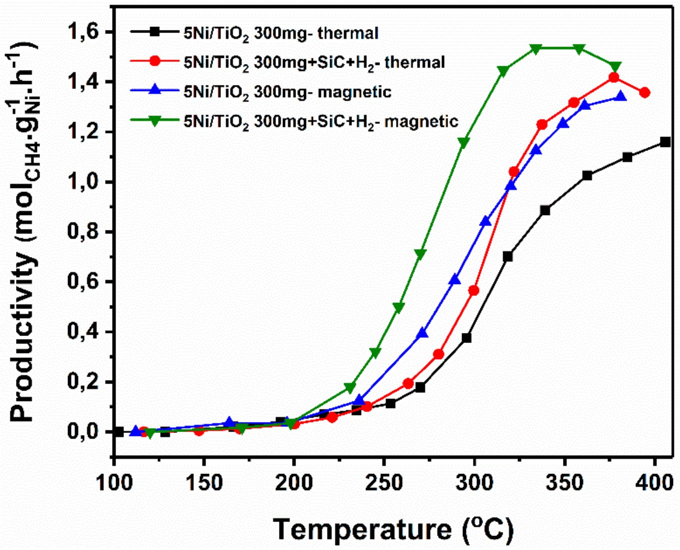

Figure S5. Productivity with respect to the reaction temperature.

Table S2. Productivity of the 5Ni/TiO2 under different reaction condition

Catalyst	%CO2 conversion	Temp. (˚C)	Productivity ($mol_{CH_4}.g_{Ni}^{-1}.h^{-1}$)
300 mg (thermal)	24.2	300	0.43
300 mg (magnetic)	42	300	0.75
300 mg + H2 + SiC (thermal)	31.9	300	0.57
300 mg + H2 + SiC (magnetic)	69.3	300	1.24
300 mg (thermal)	47.1	335	0.84
300 mg (magnetic)	62.8	335	1.12
300 mg + H2 + SiC (thermal)	66.4	335	1.19
300 mg + H2 + SiC (magnetic)	85.4	335	1.52

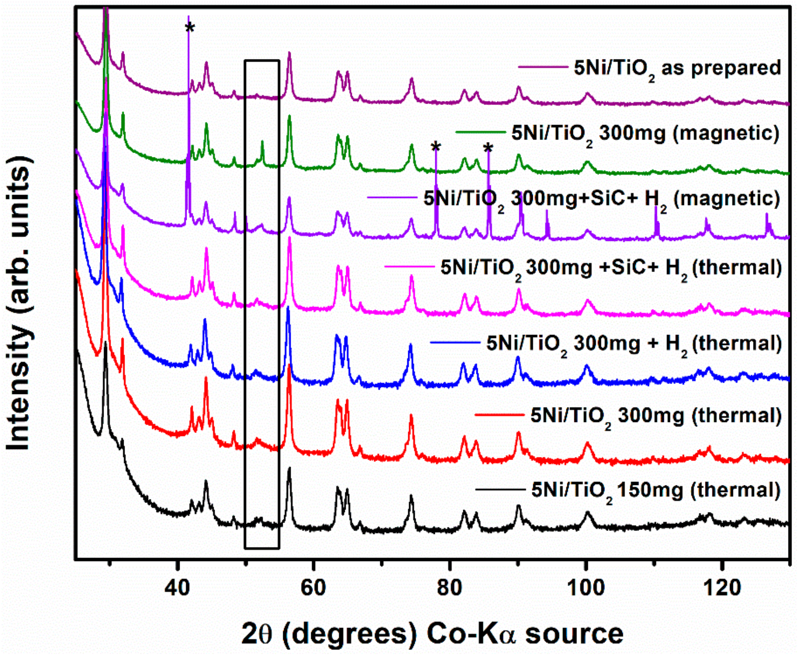

Figure S6. Stack plot of powder XRD patterns of as prepared and post catalytic samples. The diffraction peaks of SiC are marked in asterisk. The region of interest containing diffraction peak correspond to the (111) lattice plane of *fcc* phase of Ni is marked in black box.

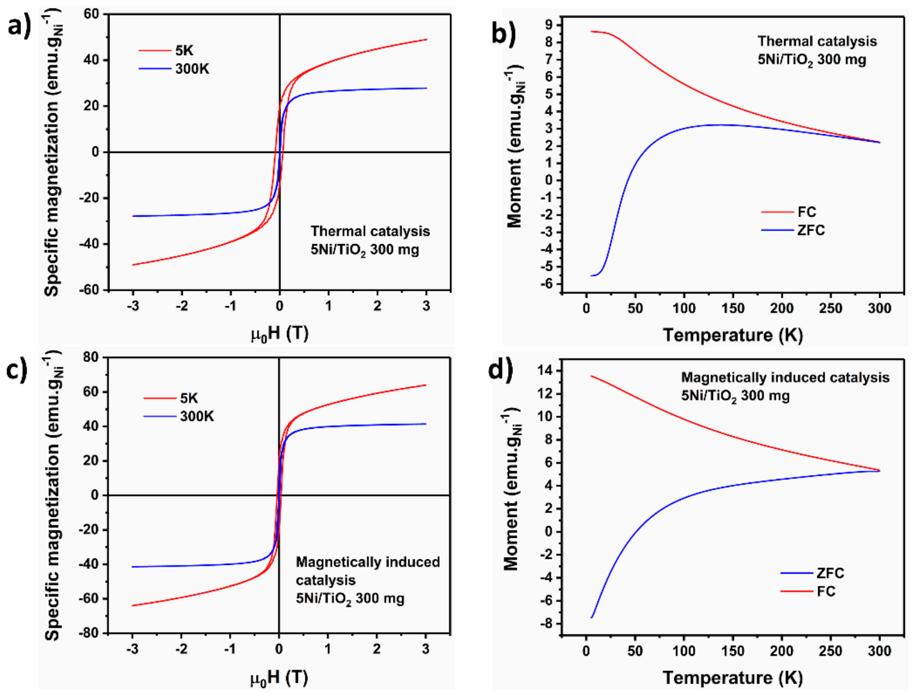

Figure S7. a,c) M-H curve measured at 5 (red curve) and 300 K (blue curve), b,d) ZFC (zero field cooling, blue trace) and FC (field cooling, red trace) curve plotted against the temperature. 300 mg of 5Ni/TiO_2 catalyst after the catalytic test carried out under thermal (a,b) and magnetic induction heating (c,d).

Table S3. Saturation magnetization and coercive field of as prepared and post catalytic 5Ni/TiO_2 sample

**Sample**	**M_s (emu.g_{Ni}^{-1}) at 300 K**	**M_s (emu.g_{Ni}^{-1}) at 5 K**	**H_c (Oe) at 5 K**
As prepared 5Ni/TiO_2	9.4	18.3	238
300 mg 5Ni/TiO_2 (thermal)	27.8	35.7	832
300 mg 5Ni/TiO_2 (magnetic)	41.4	47.2	410

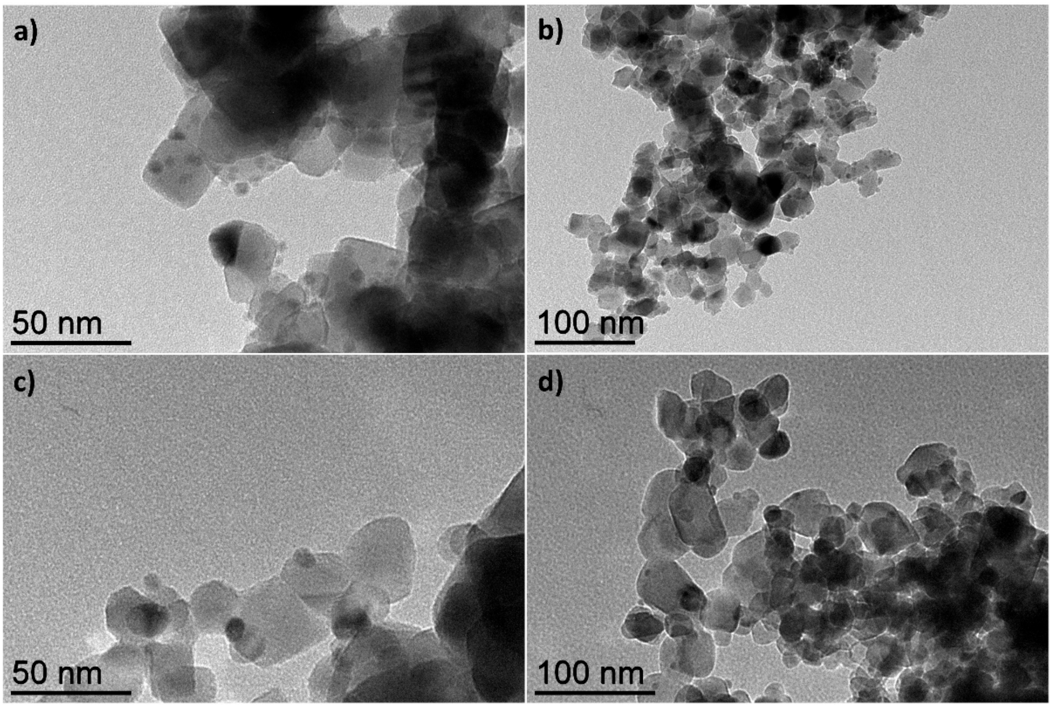

Figure S8. BF TEM images of $5Ni/TiO_2$ catalyst after the 45 h of stability test, a,b) under classical heating, and c,d) under magnetic induction heating.

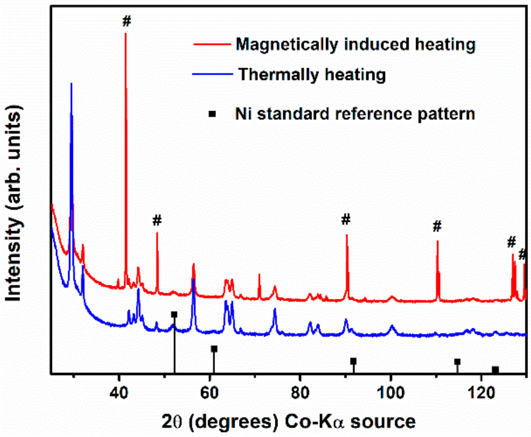

Figure S9. Overlay of powder XRD patterns of spent catalyst obtained after 45 h of stability test under thermal (blue) and magnetically induced heating condition. The diffraction peaks for SiC was marked as # symbol. The standard diffraction pattern for bulk Ni was marked as black vertical droplines (ICDD: 00-004-0850 for *fcc* Ni). It was found that the diffraction peaks correspond to the Ni are broad. The crystallite size was not calculated using the Debye-Scherrer equation. Hence, no conclusion was made from this study.

References

1. Chou, C.-Y. ; Loiland, J.A.; Lobo, R.F. *Catalysts* **2019**, *9(9)*, 773.

2. Loiland, J.A.; Wulfers, M.J.; Marinkovic, N.S.; Lobo, R.F. *Catal. Sci. Technol.* **2016**, *6*, 5267-5279.
